# Big data phenotyping in rare diseases: some ethical issues

**DOI:** 10.1038/s41436-018-0067-8

**Published:** 2018-06-15

**Authors:** Nina Hallowell, Michael Parker, Christoffer Nellåker

**Affiliations:** 1 Big Data Institute, University of Oxford, Oxford, UK; 2 Ethox Centre, Nuffield Department of Population Health, University of Oxford, Oxford, UK; 3 Wellcome Centre for Ethics and Humanities, University of Oxford, Oxford, UK; 4 Nuffield Department of Obstetrics and Gynaecology, University of Oxford, Oxford, UK

Phenotyping based on the analysis of photographic images is refining the categorization of rare genetic disorders.^1^ Through the development of facial recognition technology incorporating machine learning algorithms (MLAs) this big data approach to phenotyping—computational phenotyping—provides statistical support for determining causative variations and enables patient “matchmaking” for ultrarare or currently unknown disorders.^2^

Computational phenotyping is a “promissory” technology. From the patient/family perspective it promises a shortened clinical pathway to diagnosis, and the potential for noninvasive treatment monitoring and/or progressive risk assessment. Computational phenotyping provides diagnostic support tools for clinical geneticists and enables genomics researchers to identify new syndromes through precise and comprehensive characterization of phenotypes, facilitating the identification of novel patterns and similarities.^2^ The development of this technology supports precision medicine initiatives^3^ through further stratification of rare disease phenotypes and, because it may produce faster and more accurate diagnoses, offers public and private healthcare systems potentially reduced healthcare costs. Finally, it enables private and public institutions to leverage profit through the commercialization of phenotyping tools and training datasets.

Although there are many benefits and beneficiaries of computational phenotyping, its use raises a number of ethical and legal issues. Some of these pertain to the use of personal data in general and have been well documented, namely, the challenges of achieving valid consent for data use and protecting confidentiality, and addressing threats to privacy, data protection, and copyright.^4^ These issues are particularly challenging in computational phenotyping research in rare diseases, as this often involves the use of image (i.e., identifiable) data from children.^5^ While issues of data ownership, data security, and data access^6^ are important, other ethical issues generated by the use of image and other digital data in computational phenotyping have been described.^7^ In this paper we discuss three of these, which we believe are relevant to computational phenotyping: data-induced discrimination, the management of incidental findings, and the commodification of (phenotypic) datasets. All apply to the use of MLAs in general,^7–9^ and their use in other healthcare contexts, and will become more relevant for those working in genetics research and clinical practice as computational phenotyping tools are increasingly deployed.

## The potential for data-induced discrimination

The first is the potential for MLAs to develop algorithmic bias, which may lead to social discrimination and result in inequitable access to healthcare. The algorithms used in computational phenotyping incorporate inductive methods to detect associations between, or patterns within, datasets. The diagnostic accuracy and informative value of the resulting phenotyping tools is therefore, determined by the amount and quality—the volume, variety, and veracity—of data used in model training. Thus, the success of computational phenotyping in rare diseases depends on compiling a representative database of photographic facial (or other) images plus diagnostic and other phenotypic and/or genotypic information for algorithm training. Training data may be procured from two sources: from clinicians/researchers through data-sharing consortia (e.g., the Minerva Consortium) and directly from patients (e.g., Minerva & Me https://www.minervaandme.com).

Methods of data procurement can induce bias in MLAs where the resulting training sets are too homogeneous and fail to reflect real world diversity. This problem is particularly pertinent in the case of computational phenotyping for rare disease because MLAs need to be able to distinguish disease from nondisease-related phenotypes, and can only do so if exposed to a wide spread of phenotypic variation. Furthermore, because there are strong influences of genetic ancestry on facial characteristics, which are unrelated to disease-related phenotypes, the underrepresentation of individuals of non-European ancestry in facial phenotyping is potentially problematic.^10^ So to maximize their clinical utility and avoid algorithmic bias, computational phenotyping projects must ensure the curation of ethnically diverse training sets. However, recruiting different ethnic groups to these projects can be challenging, partly because of lack of resources, partly because in some contexts genetic disorders may be perceived as stigmatizing and partly because photographic data may be regarded as sensitive in some cultural groups.^5^ A failure to ensure the equitable representation of diverse populations in computational phenotyping initiatives will create biased tools that fail to ignore ancestral background and may result in inequitable access to this technology across global settings. While it may be difficult to eradicate these biases completely, they can, and should, be acknowledged. Developers of phenotyping tools should ensure they are aware of the demographic makeup of the training datasets they use and provide this information for clinicians and other users.

## Incidental findings: an outcome of the use of inductive methods

Combining differing datasets containing sensitive personal information (e.g., digitized facial images, genomic and clinical information) may result in unexpected (co)incidental findings (IFs), which are unrelated to the primary research or clinical question (e.g., false paternity, drug usage, or somatic disease phenotypes). IFs may result from the MLAs’ capacity to consider many different patterns within the combined dataset simultaneously and, as a result, return phenotypic patterns that were not originally sought. For example, rare disease phenotyping tools will have to be trained to “ignore” coincidental traits in facial images that are indicative of other diseases or conditions, such as Cushing’s disease, polycystic ovarian syndrome, hepatitis, or alcohol abuse, all of which have associated changes in skin tone and/or facial appearance.

Ethical issues arising from the generation of IFs are not new. There has been a great deal of discussion about clinicians’ and researchers’ obligations to disclose IFs (i.e., additional/secondary findings) in next-generation sequencing (NGS)^11^ (and medical imaging^12^). Arguably, however, IFs generated by MLAs from diverse and previously unrelated datasets differ from those produced in NGS because they are likely to be genuinely unexpected and novel in many cases—indeed, this is the point of using MLAs. In NGS, in contrast, even findings that are “incidental” are somewhat “predictable” because they depend upon prior decisions about which areas of the genome are targeted for interpretation.

The fact that MLAs may produce IFs is not ethically neutral, for this is an unintended consequence of algorithm design and prior decisions about the selection of datasets, which in turn raises ethical questions, such as how should these decisions be reviewed and evaluated when MLAs are essentially black boxes, how should accountability and responsibility be managed, and if IFs are a likely consequence of MLA-driven approaches, is there a duty to disclose this information to research participants/patients? It seems likely that the nature and scope of the duty to disclose IFs will differ depending on whether these phenotyping tools are deployed in research or clinical practice, activities characterized by different ethico-legal relationships, duties, and obligations.^11^

## Anticipatory value and the commodification of phenotypic data

The construction of data as “exploitable raw materials”;^13^^:4^ that can be endlessly repurposed suggests that digital data, like biosamples and electronic healthcare records, are potentially an important resource. In this sense, big data methods can be seen as creating new forms of value—anticipatory value. The anticipatory value of different data types derives in part from all of the future uses to which they may be put, which in turn reside in the potential relationships constructed during data mining or analysis.

Anticipatory value is not just a form of performative value, but also relates to the economic opportunities that data afford.^14^ Private companies, nation states, public institutions (health systems, universities, biobanks), and academic researchers are increasingly aware of the value accumulating within big datasets, with the result that the medical and societal potential of computational phenotyping tools are threatened, because the datasets on which they rely are increasingly co-opted by commercial or academic interests. This raises a number of ethical concerns, including a lack of ethical oversight of data use in private corporations, the need for impartial documentation of clinical utility, equity questions in the control and use of data, further commodification of personal information and attempts to monopolize data access. The latter is perhaps the most important, as restricting access to datasets undermines public interest in “…the development of knowledge and innovation through scientific research.”^10:P6^ Indeed, the quality of phenotyping and utility of phenotyping tools is directly related to the quality of the datasets used for algorithm training, therefore, the siloing of datasets and restricting data access potentially inhibits machine learning and may result in biased outputs from MLAs. To prevent data-siloing and ensure that all can benefit from these technologies, we need to start treating geno-/phenotypic and other digital health data as public goods rather than private resources. This will necessitate new forms of regulation and data governance structures to ensure those who curate and control datasets act in the wider public interest.

## Conclusions

In conclusion, the development of computational phenotyping has the potential for transformative health (and other societal) benefits. However, this technology raises a number of ethical questions that need to be addressed if these benefits are to be fully realized. These include: how should we avoid the potential for algorithmic bias and data-induced discrimination, how should IFs be managed, and what should we do about the increasing commodification of datasets, which may compromise the development of this technology for the public good?
